# Colloid Carcinoma of the Extrahepatic Biliary Tract with Metastatic Lymphadenopathy Mimicking Cystic Neoplasm: A Case Report

**DOI:** 10.5812/iranjradiol.7234

**Published:** 2013-05-20

**Authors:** Na Yeon Han, Beom Jin Park, Deuk Jae Sung, Min Ju Kim, Sung Bum Cho, Dong Sik Kim, Jeong Hyeon Lee

**Affiliations:** 1 Department of Radiology, Anam Hospital, Korea University College of Medicine, Seoul, Korea; 2 Department of Surgery, Anam Hospital, Korea University College of Medicine, Seoul, Korea; 3 Department of Pathology, Anam Hospital, Korea University College of Medicine, Seoul, Korea

**Keywords:** Adenocarcinoma, Mucinous, Bile Ducts, Extrahepatic, Lymphatic Diseases, Ultrasonography

## Abstract

The patient is a previously healthy 52-year-old woman who presented with dyspepsia for two months. Multiple imaging modalities including ultrasound, computed tomography (CT), and magnetic resonance imaging (MRI) showed diffuse bile duct dilatation with an obstructive lesion of the distal extrahepatic biliary duct (EHD) as well as two masses in the peripancreatic area. The peripancreatic masses appeared cystic with posterior acoustic enhancement on ultrasound, low density on CT imaging, and high signal intensity on T2-weighted MRI. The lesion in the distal EHD exhibited similar characteristics on CT and MRI. A Whipple procedure was performed and histological specimens showed malignant cells with large mucin pools that was consistent with a diagnosis of colloid carcinoma of the EHD with metastatic lymphadenopathies.

Colloid carcinoma, also called mucinous carcinoma, is classified as a histologic variant of adenocarcinoma. Because the colloid carcinoma of the biliary tree is exceedingly rare, the imaging characteristics and the clinical features of colloid carcinoma remain relatively unknown. We report a case of colloid carcinoma of the common bile duct and its accompanied metastatic lymphadenopathies with characteristic imaging findings reflecting abundant intratumoral mucin pools.

## 1. Introduction

According to the 4th World Health Organization (WHO) classification, colloid carcinomas are newly classified as a histological variant of adenocarcinoma of the extrahepatic biliary tract (1). While commonly seen in organs such as the gall bladder and gastrointestinal tract, colloid carcinoma, also called mucinous adenocarcinoma, mucinous non-cystic carcinoma, and mucoid carcinoma, is rarely seen in the extrahepatic biliary duct (EHD).

By convention, colloid carcinomas are defined as having abundant extracellular mucin accounting for more than half of the tumor volume (2). While several previous reports have described ‘mucinous cholangiocellular carcinoma of the liver’ or ‘mucin producing bile duct tumor arising from the intrahepatic duct’, there have been no reports of colloid carcinoma of the EHD in the radiologic literature (3, 4). Colloid carcinomas are different from other mucin hypersecreting tumors such as intraductal papillary neoplasms, mucinous cystic neoplasms of the biliary tract, and ductal adenocarcinomas. It may be important to recognize this distinct subtype for optimal patient management and therapeutic planning.

We report a rare case of colloid carcinoma of the distal EHD involving the ampulla of Vater (AOV) with metastatic lymphadenopathies, including detailed imaging findings that have unique features that mimic incidental cystic neoplasm.

## 2. Case Presentation

A 52-year-old female presented with dyspepsia for two months. Her past medical history was unremarkable, and there were no significant findings on physical examination. On initial ultrasound imaging, the common bile duct was diffusely dilated with an abrupt narrowing at the distal EHD. Additionally, two elongated heterogeneous iso- to hypo-echoic masses with posterior acoustic enhancement (approximately 3.5 × 5 cm and 2.4 × 1.5 cm) were detected near the portal vein and the uncinate process of the pancreas (Figure 1). Laboratory studies, with the exception of an elevated carcinoembryonic antigen (54 ng/mL) and alkaline phosphatase (285 IU/L), were within normal limits. There was no jaundice in spite of the biliary tree dilatation, suggesting partial obstruction.

**Figure:**
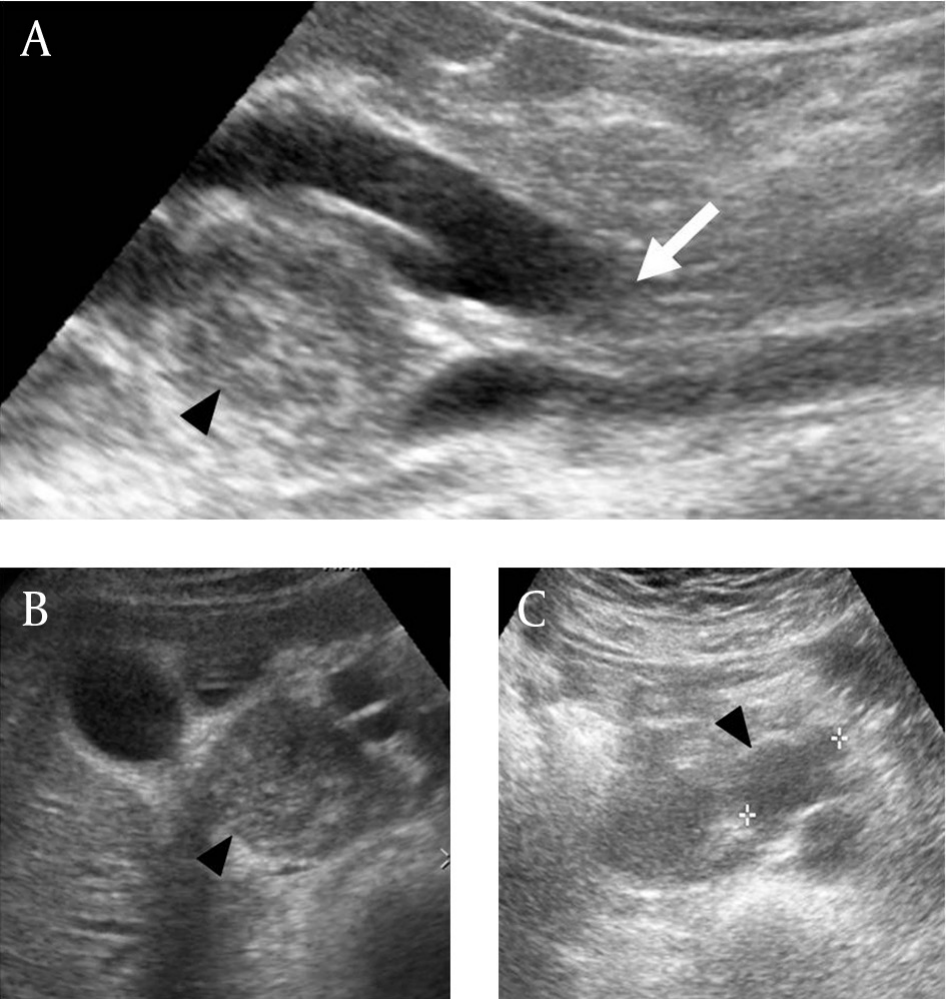
Figure 1. Abdominal ultrasonography. A, Longitudinal scan shows diffusely dilated extrahepatic bile duct with an ill-defined obstructive lesion (white arrow) in the distal portion and heterogeneous echoic mass (arrowhead) adjacent to the EHD. B, Axial scan shows the mass seen in A (arrowheads) with intense posterior enhancement along the hepatoduodenal ligament between the EHD, cystic duct, and gall bladder. C, Axial scan shows another hypoechoic mass posterior to the uncinate process of the pancreas.

The 4 channel multidetector computed tomography (MDCT) was performed and the CT demonstrated a central prominent low attenuating nodule (1.5 × 1.4 cm) with thick peripheral enhancement in the distal EHD as well as papillary bulging. Two additional cyst-like lesions were detected along the hepatoduodenal ligament posterior to the uncinate process of the pancreas (Figure 2). The mass along the hepatoduodenal ligament extended from the pancreaticoduodenal groove to the hepatic hilum with a unique drumstick-like appearance. 1.5T MRI was done and these lesions showed relatively high signal intensity on T2-weighted imaging with intervening linear low signal intensity similar to that seen in multi-cystic lesions (Figure 3). The pre-operative assumption was a diagnosis of cancer of the distal EHD with two incidental cystic neoplasms, possibly lymphangiomas or unusually growing pancreatic cystic neoplasms with an exophytic growth pattern. Endoscopy showed a hyperemic mass protruding through the AOV.

**Figure:**
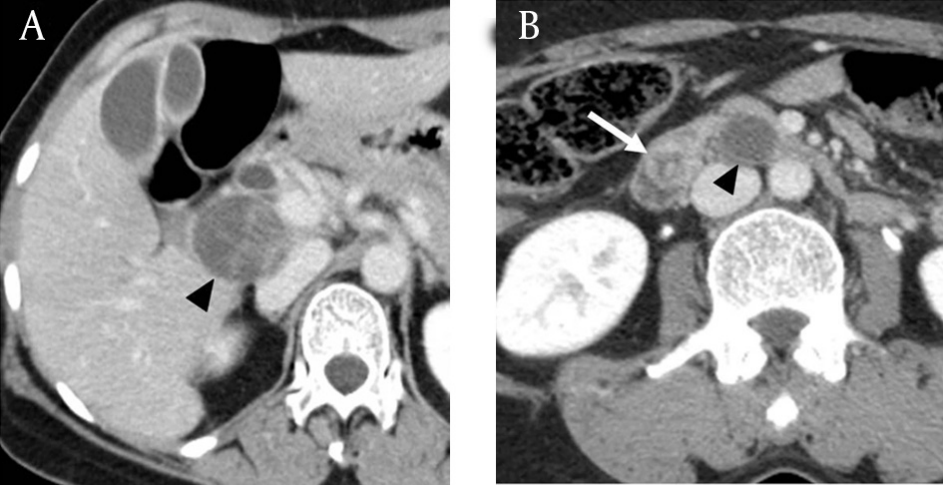
Figure 2. Portal venous phase on MDCT. A, Axial scan shows a cystic mass-like lesion with internal irregular septa along the hepatoduodenal ligament (arrowhead), misdiagnosed as benign incidental cystic neoplasm of the retroperitoneal space or the head of the pancreas. B, Axial scan at the level of the ampulla of Vater shows a nodular lesion (white arrow) with suspected central necrosis at distal EHD, and a cystic mass-like lesion abutting the uncinate process of the pancreas mimicking a benign pancreatic cystic neoplasm (arrowhead).

**Figure:**
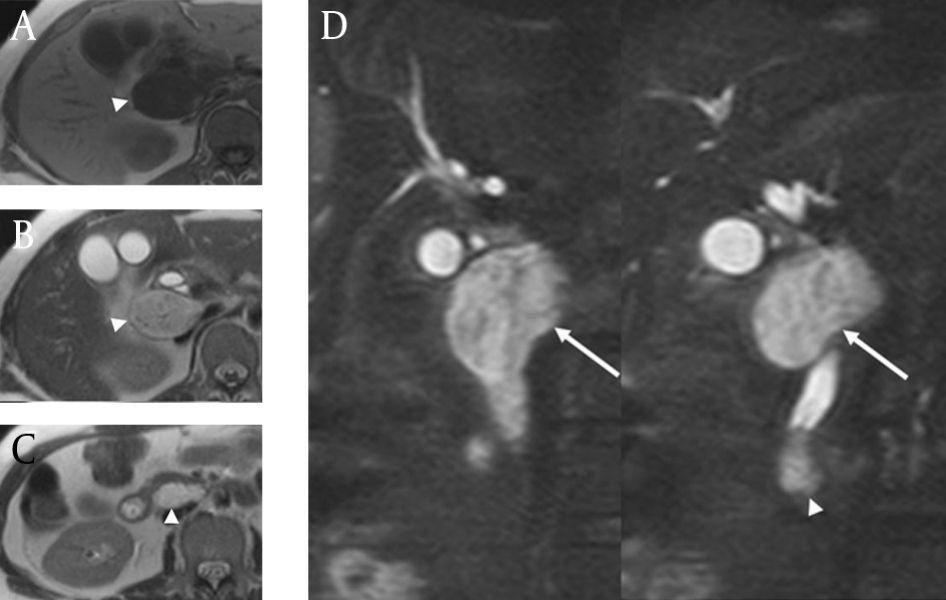
Figure 3. T2-weighted (half-Fourier acquisition single-shot turbo spin-echo, HASTE) images on 3T MRI. A, Axial T1-weighted image shows a homogeneous hypo-intense mass (arrowhead) in the portocaval area. B, C, Axial images show well-circumscribed heterogeneous hyper-intense masses (arrowheads) in the portocaval area and behind the uncinate process of the pancreas. D, Coronal images show the elongated mass seen in A (arrows) extending from behind the primary EHD lesion with internal hypo-intense septa positioned along the hepatoduodenal ligament. The primary lesion of the distal EHD also exhibited higher internal signal intensity (arrowhead) than typically seen in adenocarcinoma.

A Whipple procedure was performed successfully. The gross specimen appeared to be an irregularly shaped mass with a myxoid and hemorrhagic appearance in the distal EHD (Figure 4). The two masses appeared to be well encapsulated lesions, also with myxoid and hemorrhagic components. Microscopically, the malignant cells comprised tubular or glandular structures, and an abundance of signet ring cells were scattered in large mucin pools in both the EHD tumor and the lymph nodes. Mucinous pools were surrounded by fibrous septa. The final histopathological diagnosis was colloid carcinoma originating from the EHD with duodenal invasion and lymph node metastases. The patient had an uncomplicated postoperative course. The patient had undergone adjuvant chemotherapy using 5-fluorouracil for six cycles and serum CEA level was normalized (4.9 ng/ml, reference level <5 ng/ml) about one month after surgery. An annual follow-up CT scanning was performed and five years later, there was no evidence of tumor recurrence or distant metastasis.

**Figure:**
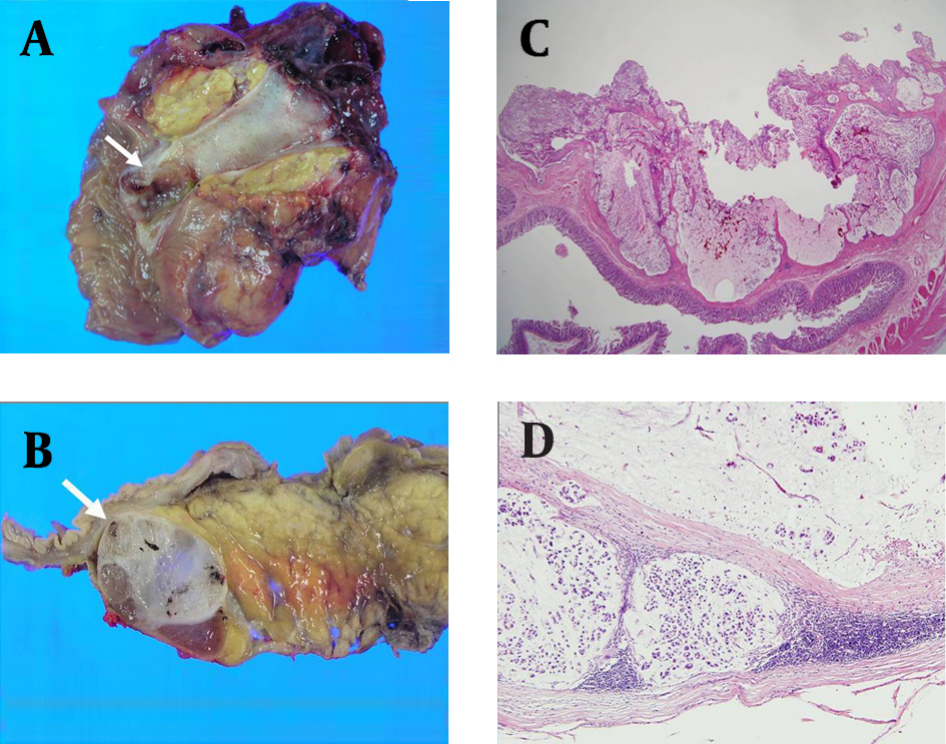
Figure 4. Photograph of the surgical specimen. A, The gross specimen shows an irregularly shaped mass in the distal EHD (arrow). B, Relatively well-demarcated soft myxoid masses with fibrous septa are noted along the serosal surface of the duodenum (arrow) and posterior to the uncinate process of the pancreas (not shown). C, Microscopic examination of a hematoxylin-eosin stained section shows predominant pools of extracellular mucin in which the neoplastic cells were suspended. The cells were primarily arranged in nests or strips, with occasional free floating cells. D, Microscopic features of metastatic colloid carcinoma in a lymph node. Mucinous lakes are surrounded by fibrous septa within the cyst. Residual lymphoid tissue is also seen in the subcapsular area, which suggests metastatic colloid carcinoma of the lymph nodes.

## 3. Discussion

Histologically, colloid carcinoma is defined by scant neoplastic cells floating in lakes of extracellular stromal mucin, constituting more than 50% of the tumor volume. In the digestive tract, it is relatively common in the stomach and colon with poor prognosis, while infrequent in the pancreas with better prognosis than ductal adenocarcinoma (5). It is very rarely seen in the biliary tract with a 0.8% reported incidence in autopsied cholangiocarcinoma cases as described in ‘mucinous intrahepatic cholangiocarcinoma’ by Nakajima et al. in 1988 (4). The clinical and imaging features of colloid carcinoma of the EHD have not yet been described due to rarity of this subtype.

Colloid carcinoma is different from mucin hypersecreting tumors, which include intraductal papillary neoplasms or mucinous cystic neoplasms of the biliary tract and pancreas. Intraductal papillary neoplasms commonly produce a large amount of mucin, which is secreted into the bile, and are identified radiologically as intraluminal polypoid lesions with biliary dilatation (6). Mucinous cystic neoplasms are multiloculated and contain mucinous or serous fluid, and are lined by columnar epithelium similar to that seen in the bile duct or foveolar gastric epithelium (7). In these tumors, excessive mucin produces cystic lesions without marked biliary dilatation because they are infrequently contiguous with the bile duct.

Characteristic histological features of colloid carcinoma in the extrahepatic biliary tree are identical to the ones described in our case. It is characterized by the presence of mucin lakes that contain scant clusters of floating cancerous cells. As seen in this case, some may show signet ring cells within the mucin pools as well (8).

There are several reports of imaging findings of colloid carcinoma of the bile duct not originated from the EHD. Hayashi et al. described radiologic characteristics of two cases of mucinous type intrahepatic cholangiocellular carcinoma, including large mucinous lakes throughout the tumor without mucin excretion into the bile duct (3, 9). Mizukami et al. (4) reported a unique presentation of mucinous carcinoma, most likely originating from the right hepatic duct. CT demonstrated multiple cystic lesions distributed adjacent to the portal area with a beaded appearance. This so called ‘periportal collar,’ reflects the tendency to form mucus lakes along the portal area (4). Despite similar histological features, differences in imaging findings between studies may be due to differences in the tumor origin and the mode of spread (4). However, abundant mucin throughout the tumor was consistently reported in each of these studies and was also noted in the present case.

In this case, the metastatic lymphadenopathies appear heterogeneously iso- to hypoechoic with posterior acoustic enhancement on ultrasound. This is likely due to the excessive mucinous component and fibrous multi-septation. Significant hypodensity on CT scan and hyperintensity on T2-weighted MR reflect abundant mucin within the tumors. T2-weighted MR also showed intervening linear low signal intensity that made the lymphadenopathy observed in this case concievably mistaken for multiseptated cystic masses. These imaging findings are consistent with reported MRI findings of colloid carcinoma of the pancreas and breast (10, 11). Pancreatic colloid carcinoma showed mesh-like low signal intensity and tiny hypointense foci, a so called salt-and-pepper appearance on T2-weighted MR reflecting the fine stroma and neoplastic cells. MRI of mucinous carcinoma of the breast also showed that secondary pathologic change such as fibrosis or hemorrhage can complicate hyperintensity on T2-weighted images (11). We believe that linear low signal intensity in this case reflected neoplastic cells, fibrosis, and hemorrhage.

In addition, the unique drumstick-like appearance of lymphadenopathy located along the hepatoduodenal ligament may be related to the ‘plastic nature’ of the tumor due to copious intratumoral mucin production. This appearance is akin to the aforementioned ‘periportal collar,’ forming mucinous lakes along the portal vein (4).

Treatment for adenocarcinoma of the EHD is common regardless of the pathologic subtype including colloid adenocarcinoma (12). In the resectable cases, surgical procedures are performed with or without adjuvant chemotherapy. In the unresectable cases, chemotherapy with supportive care is the treatment of choice.

In conclusion, colloid adenocarcinoma of the EHD with metastatic lymphadenopathy should be considered in the differential diagnosis when masses insinuating into surrounding spaces with a high mucin content and internal septum-like structures are seen on imaging in a patient with presumed common bile duct cancer.
